# Characterization of a Reservoir-Style Implant for Sustained Release of Tenofovir Alafenamide (TAF) for HIV Pre-Exposure Prophylaxis (PrEP)

**DOI:** 10.3390/pharmaceutics11070315

**Published:** 2019-07-04

**Authors:** Leah M. Johnson, Sai Archana Krovi, Linying Li, Natalie Girouard, Zach R. Demkovich, Daniel Myers, Ben Creelman, Ariane van der Straten

**Affiliations:** 1 Engineered Systems, RTI International, 3040 E. Cornwallis Road, Research Triangle Park, NC 27709, USA; 2 Women’s Global Health Imperative, RTI International, 351 California Street, Suite 500, San Francisco, CA 94104, USA; 3 PATH, 2201 Westlake Ave, Suite 200, Seattle, WA 98121, USA

**Keywords:** poly(ε-caprolactone) (PCL), tenofovir alafenamide (TAF), pre-exposure prophylaxis (PrEP), long-acting drug delivery systems, implant

## Abstract

Long-acting (LA) HIV pre-exposure prophylaxis (PrEP) offers the potential to improve adherence by lowering the burden of daily or on-demand regimens of antiretroviral (ARV) drugs. This paper details the fabrication and in vitro performance of a subcutaneous and trocar-compatible implant for the LA delivery of tenofovir alafenamide (TAF). The reservoir-style implant comprises an extruded tube of a biodegradable polymer, poly(ε-caprolactone) (PCL), filled with a formulation of TAF and castor oil excipient. Parameters that affect the daily release rates of TAF are described, including the surface area of the implant, the thickness of the PCL tube walls (between 45 and 200 µm), and the properties of the PCL (e.g., crystallinity). In vitro studies show a linear relationship between daily release rates and surface area, demonstrating a membrane-controlled release mechanism from extruded PCL tubes. Release rates of TAF from the implant are inversely proportional to the wall thickness, with release rates between approximately 0.91 and 0.15 mg/day for 45 and 200 µm, respectively. The sustained release of TAF at 0.28 ± 0.06 mg/day over the course of 180 days in vitro was achieved. Progress in the development of this implant platform addresses the need for new biomedical approaches to the LA delivery of ARV drugs.

## 1. Introduction

HIV pre-exposure prophylaxis (PrEP) with antiretroviral (ARV) drugs is promising among the biomedical strategies to address the global HIV epidemic. Tenofovir-based PrEP has demonstrated landmark successes with daily [1,2,3,4] and on-demand dosing [3,5] in men who have sex with men (MSM) and transgender women (TGW). Despite these advancements, adherence to time- or event-driven regimens for PrEP remains an incessant struggle [6,7,8,9,10,11]. The long-acting (LA) delivery of ARV drugs simplifies traditional dosing regimens for PrEP by alleviating the emotional and logistical burden of user-dependent methods. For example, a LA-injectable formulation of the integrase inhibitor, cabotegravir (CAB), is currently under investigation in a pair of phase two and three HIV PrEP trials [12,13]. Although injectable methods are acceptable to many users [14,15], and offer key advantages such as a bi-monthly dosing regimen and discretion, drawbacks do exist. Injectable formulations cannot be removed in the event of an adverse drug-related event and the potential exists for a long plasma “tail” of sub-therapeutic drug levels [16,17]. An alternative injectable formulation in preclinical development involves an in situ forming polymer depot for LA delivery of dolutegravir for HIV PrEP, which shows the capacity for removal within the period of drug delivery, if needed [18].

A promising biomedical approach for LA-PrEP involves implants that reside under the skin to continuously release the drug, which supports adherence over longer time periods, enables discretion of use, lowers the burden of the regimen, and remains reversible during the therapeutic duration. Polymeric implants can comprise different architectures that each have advantages for drug delivery [19,20,21]. Matrix-style implants contain a drug dispersed within a polymer that controls the rate of the drug exiting the implant. For example, an FDA-approved matrix-style implant for the six-month maintenance treatment of opioid addiction (Probuphine^®^) contains buprenorphine distributed through four individual poly(ethylene-vinyl acetate) (EVA) rods [22]. Reservoir-style implants involve a formulated drug core encapsulated by a polymeric barrier that control drug release rates. Notable examples of implants with a core-sheath configuration include the collection of subdermal contraceptive implants: Norplant and Jadelle [23,24] for the delivery of levonorgestrel (LNG) using a rod of silicone-based polymer, as well as Implanon [25] and Nexplanon [26] for the delivery of etonogestrel (ENG) using a rod of EVA-based polymer. The low dosages required for the subcutaneous delivery of hormonal contraceptives enable these implants to last multiple years. Implants also show utility for indications in ophthalmology, including intraocular implants for the delivery of ganciclovir for the treatment of cytomegalovirus retinitis (Vitrasert) [27], dexamethasone for the treatment of macular edema (Ozurdex) [28,29] and fluocinolone acetonide for the treatment of noninfectious posterior uveitis (Retisert) [30].

Several implants are currently under development for HIV PrEP, with each implant system holding unique configurations and features. A subdermal, silicone implant that delivers tenofovir alafenamide (TAF) from orthogonal channels coated with polyvinyl alcohol (PVA) showed 40-days of drug delivery in beagle dogs without observed adverse events [31]. A non-polymeric, refillable implant designed to deliver TAF and emtricitabine (FTC) from separate devices showed sustained levels of tenofovir diphosphate (TFV-DP) in peripheral blood mononuclear cells (PBMCs) over 83 days in rhesus macaques, but only 28 days for FTC-triphosphate (FTC-TP) due to the large dosing required and short plasma half-life [32]. Intarcia is developing a titanium osmotic pump system, called the Medici Drug Delivery System™, for PrEP and for type-2 diabetes [33]. A matrix-style PrEP implant for delivery of 4′-ethylnyl-2-fluoro-2′-dexoyadenosine (EFdA) has shown promising efficacy for HIV treatment and prevention, as demonstrated in animal models [34].

Concurrently with these other innovative technologies, RTI is developing a subcutaneous biodegradable implant for HIV PrEP as a single indication and as a multipurpose prevention technology (MPT) for HIV and pregnancy prevention [35,36]. The implant uses a semi-crystalline aliphatic polyester, poly(ε-caprolactone) (PCL), pioneered by Pitt et al. at RTI International in the 1980s [37] and largely neglected for nearly 20 years [38]. Renewed appeal for PCL has surfaced in light of biomedical applications, including tissue engineering [39,40] and drug delivery [41,42], that demand materials with long-term functionality, mechanical integrity, biocompatibility, and capacity for biodegradation and bioresorption. PCL is currently used in FDA-approved products for root canal fillings (Resilon) [43] and sutures (Monocryl) [44] and was previously explored for use as a 1-year contraceptive implant (Capronor) [45]. In terms of HIV PrEP, PCL implants can advantageously offer LA delivery of ARVs, while also enabling bioresorption at the end of the implant drug delivery period. An implant that is biodegradable could benefit health care systems by eliminating the need for a clinic visit to remove a depleted implant, whereby a minor surgical procedure would be required to remove the implant when discontinuing PrEP. In the case of the implant described in this paper, reversibility and retrievability can be maintained, potentially throughout the duration of treatment.

Herein, we report advancements to the fabrication and performance of a reservoir-style subcutaneous PCL implant for sustained release of TAF. We detail parameters that control release rates of TAF from the implant, including wall thickness and surface area, and further describe the effects of crystallinity on the performance of the implant. We demonstrate the fabrication and processing steps that align the implant with future manufacturing requirements, while keeping preferences of the end user in mind.

## 2. Materials and Methods

### 2.1. Implant Fabrication

PCL pellets were purchased in research-grade from Sigma Aldrich, referred to as “Sigma-PCL” throughout this paper (number average molecular weight (*M*_n_) = 103 kDa, Cat# 440744, St. Louis, MO, USA) and in medical-grade from Corbion, referred to as “PC-12” throughout this paper (*M*_n_ = 51 kDa, PURASORB PC 12, Amsterdam, The Netherlands). PCL tubes were fabricated via a hot-melt, single screw extrusion process using solid PCL pellets at GenX Medical (Chattanooga, TN, USA). All tubes were 2.5 mm in outer diameter (OD) and had wall thicknesses of 45, 70, 100, 150 or 200 µm, as measured with a 3-axis laser measurement system and light microscopy at GenX Medical.

PCL tubes were first sealed at one end using two different approaches: impulse heat sealing and injection sealing. Implants fabricated with the impulse heat sealing were used for certain in vitro studies, such as the implants used in the surface area studies that comprised different lengths (e.g., 70 mm length), because they were too long to fit the injection sealing apparatus. Importantly, no significant differences in release rates were observed for TAF implants sealed with either approach. For the first approach, an impulse heat sealer (AIE-110T, American International Electric Sealer Supply, South El Monte, CA, USA) was used to clamp the tube flat and then apply a pulse of heat for a few seconds. The tubing was then allowed to cool for about 10 s. Thicker tubes were sealed with longer heat pulses. The sealing step fused the PCL tube wall together through melting and created a flat-shaped seal. The seal was trimmed with scissors to remove excess PCL. For the injection sealing, the PCL tube was marked and trimmed to the correct length to achieve an implant with a 40-mm paste length with 3 mm of headspace at both ends for sealing. The initial seal was then created on one end of the implant by placing the tube over a stainless steel rod that filled all the tube except for a 3 mm headspace at one end, placing a Teflon collar around the headspace to support the tube wall and injecting molten PCL into the cavity of the headspace. After the injected PCL was solidified, excess PCL was trimmed, and the collar was removed to form a cylindrical seal approximately 2 mm long that is compatible with commercial contraceptive trocars. 

TAF was graciously provided by Gilead Sciences (Foster City, CA, USA). TAF was mixed with pharmaceutical grade, Super Refined^TM^ Castor Oil (Croda, Cat# SR40890, Snaith, UK) at 2:1 mass ratio immediately prior to loading into the implant. The mixture was first ground with a mortar and pestle to create a smooth paste, and then back loaded into a 1 mL syringe fitted with a 14-gauge blunt tip needle. The TAF and castor oil paste was then extruded through the needle into the empty tube. Otherwise, the TAF formulation was loaded into the PCL tube using a modified spatula. After the filled formulation reached the 40-mm mark, the interior tube wall was cleaned with a rod and sealed in a similar manner to the first seal. After fabrication, all devices were weighed to determine the total payload and photographed with a ruler to record the final dimensions. Paste area was measured with ImageJ (Version 1.50e, NIH, Bethesda, MD, USA) and release rates were normalized to the surface area of a full-sized implant (2.5 mm OD, 40 mm in length), 314 mm^2^. The end of the implants (i.e., end-seals) were not included in calculations of the implant surface area.

### 2.2. Device Sterilization

All implants were fabricated and handled under aseptic conditions using a biosafety cabinet. Certain devices were exposed to gamma irradiation, as indicated in the text. Devices exposed to gamma irradiation were first packed in amber glass vials and then irradiated with a dose range of 18–24 kGy at room temperature, using a Cobalt-60 gamma-ray source (Nordion Inc., Ottawa, Canada) at Steris (Mentor, OH, USA). Samples were exposed to the source on a continuous path for a period of 8 h.

### 2.3. In Vitro Release Studies

In vitro release characterization involved incubation of the implants in 40 mL 1X phosphate buffered saline (PBS) (pH 7.4) at 37 °C and placed on an orbital shaker. TAF species in the release media was measured by ultraviolet-visible (UV) spectroscopy at 260 nm using the Synergy MX multi-mode plate reader (BioTek Instruments, Inc, Winooski, VT, USA). The release buffer was sampled three times per week during which the devices were transferred to 40 mL of fresh buffer to maintain sink conditions. TAF quantity released in each PBS buffer during the time interval was calculated and cumulative mass of drug release as a function of time was determined.

### 2.4. Stability Analysis of TAF Formulation

The purity of TAF formulations inside the device reservoir was evaluated by opening a device, extracting the entire reservoir contents into an organic solution, and measuring TAF chromatographic purity using ultra performance liquid chromatography coupled with UV spectroscopy (UPLC/UV). The analysis was performed using a Waters BEH C18 column (2.1 mm × 50 mm, 1.7 μm) under gradient, reversed phase conditions with detection at 260 nm. For each device, one single aliquot was prepared and quantitated by linear regression analysis against a five-point calibration curve. TAF purity was calculated as % peak area associated with TAF relative to total peak area of TAF related degradation products (detected above the limit of detection (LOD) ≥ 0.05%). The TAF formulations within the implant were analyzed after exposure of the implant to a simulated physiological condition (i.e., 1X PBS, pH 7.4 at 37 °C) for up to 180 days.

### 2.5. Characterization of PCL Extruded Tubes

#### 2.5.1. Differential Scanning Calorimetry (DSC)

The melting behavior of PCL samples was assessed with modulated differential scanning calorimetry (MDSC) (TA Instruments Q200, RCS90 cooling system, New Castle, DE, USA). Approximately 8 mg of extruded polymer tubing was placed in a Tzero^TM^ Pan and sealed with a Tzero^TM^ Lid and a dome-shaped die, resulting in a crimped seal. Samples were then placed in a nitrogen-purged DSC cell, cooled to 0 °C, then heated to 120 °C at a rate of 1 °C/min with an underlying heat-only modulation temperature scan of ± 0.13 °C every 60 s. The melting temperature (*T_m_*) of the polymer was determined by the peak temperature of the melting endotherm, and the enthalpy associated with melting was determined by integrating linearly the area of the melt peak (between 25 and 65 °C) using the TA Universal Analysis software (version 4.5A, TA Instruments, New Castle, DE, USA). PCL samples did not exhibit exothermic peaks in the non-reversing heat flow signal indicating that PCL did not experience cold-crystallization during the melting process; therefore, the total heat flow curve was used to assess the mass % crystallinity. The mass % crystallinity was calculated using Equation (1), where *X_c_* represents the mass fraction of crystalline domains in PCL, Δ*H_m_* represents the enthalpy of melting measured by the DSC, and Δ*H_fus_* represents the theoretical enthalpy of melting for 100% crystalline PCL, reported as 139.5 J/g [46,47].

(1) $$X_{C}=\frac{\Delta H_{m}}{\Delta H_{fus}}\times 100\;$$

The peak melting temperatures of polymers were used calculate crystallite sizes within the sample using the Thompson–Gibbs equation (Equation (2)) [48,49]:

(2) $$L=\frac{2\sigma_{e}T_{m}^{o}}{\Delta H_{m}^{o}\left(T_{m}^{o}-T_{m}\right)}$$

where *L* is the crystallite size in nm, *σ_e_* is the free energy of chain folds in mJ/m^2^, $T_{m}^{o}$ is the equilibrium melting temperature in K, *T_m_* is the melting temperature measured by DSC in K, and $\Delta H_{m}^{o}$ is the enthalpy of fusion for 100% crystalline polymer in J/g. $T_{m}^{o}$ and $\Delta H_{m}^{o}$ were taken from the ATHAS data bank as 342.2 K and 139.5 J/g, respectively. The free energy associated with chain folding was taken as 60 mJ/m^2^ [50].

#### 2.5.2. X-ray Diffraction (XRD)

The extruded PCL tubes at wall thickness of 100 µm were cryo-grinded in a freezer mill using liquid nitrogen. The material was ground for 1.5 min after cooling for three minutes before initiating the grinding cycle. The X-ray diffraction (XRD) patterns were acquired using a Bruker AXS, Inc D8 Advance model utilizing standard Bragg-Brentano geometry and a LynxEye XE-T high resolution detector (Bruker, Billerica, MA, USA). Samples were packed into a zero background sample holder and scanned at 40 kV and 40 mA power settings (1600 Watts) for a scan covering 5° to 70°, with a step size of 0.02° and a dwell time of 2 s per step. The MDI Jade version 9.6 software (MDI, Livermore, CA, USA) was used to analyze results and the 2019 International Center for Diffraction Data (ICDD) PDF 4+ database was used to search match crystalline phases present in the materials. The crystallite size was determined via the Scherrer equation (Equation (3)):

(3) $$L=\frac{K\lambda }{\beta cos\theta }$$

where *L* = crystallite size, *K* = Scherrer constant (0.94 from literature [51,52]), *λ* = X-ray wavelength, *β* = full-width at half maximum of a crystallographic peak, and *θ* = Bragg angle.

#### 2.5.3. Gel Permeation Chromatography (GPC)

The molecular weight of PCL was analyzed via GPC by first dissolving samples in tetrahydrofuran (THF) to 10 mg/mL injecting 40 µL of sample using an Agilent 1100/1200 HPLC-UV instrument (Santa Clara, CA, USA, flow rate of 1.0 mL/min). Polystyrene polymer standards (MWs of 2460 to 0.545 kDa) were used to calibrate the MW of samples. 

#### 2.5.4. Statistical Analysis

Where indicated, significance testing was performed with GraphPad Prism 7.00 (GraphPad Software, San Diego, CA, USA) using an unpaired, parametric, two tailed, *t*-test with a confidence level of 95%. Probability (*p*)-values ≤ 0.05 were considered statistically significant.

## 3. Results and Discussion

### 3.1. Tuning TAF Release Rates: Surface Area and Wall Thickness

**Figure 1:**
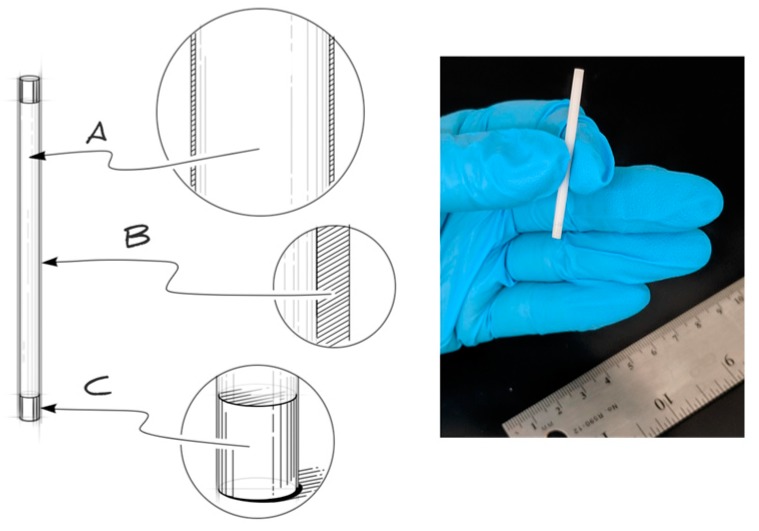
(Left) A schematic of a PCL reservoir-style device for delivery of TAF, which comprises a formulated drug core (A) encapsulated by a rate-controlling PCL membrane (B). The device is end-sealed using PCL material (C) for trocar compatibility. (Right) A digital camera image of the biodegradable implant.

These studies involve a reservoir-style PCL implant (Figure 1), that can deliver TAF at sustained, zero-order release kinetics. Once inserted subcutaneously, biological fluid from the surrounding environment transports through the PCL membrane into the reservoir and can solubilize TAF. TAF can partition into the PCL and transport passively through the PCL membrane to exit the implant. Transport of a drug through the PCL material is dictated by many parameters, such as the diffusion coefficient and partition coefficient, as described elsewhere [53]. As an aliphatic polyester, PCL undergoes bulk hydrolysis through random chain scission as water permeates through the polymer [37,54]. However, biodegradation of PCL is slow and can require years (e.g., 1–2 years) for complete bioresorption [37], depending on the starting MW. Because bulk erosion of PCL is slow, the faster process of drug delivery is decoupled from biodegradation, enabling zero-order release profiles of drug from the implant. At this zero-order release profile, the daily drug delivery rates are controlled by various parameters: surface area of the device, thickness of the device wall, polymer properties, and drug formulation. A digital camera image of RTI’s trocar-compatible reservoir-style implant is shown in Figure 1.

**Figure 2:**
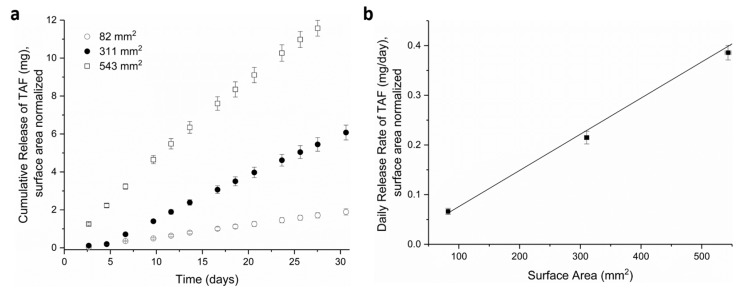
In vitro release studies showing (**a**) cumulative release of TAF from implants of differing surface areas and (**b**) daily release rates of TAF at day = 24 for implants with different surface areas. All implants were fabricated with Sigma-PCL, 100 µm wall thickness and a formulation of 2:1, TAF:castor oil. Surface areas were normalized according to the theoretical surface area for implant of 10, 40, 70 mm in length. Three implants were tested per condition.

To evaluate the relationship between release rates and the surface area of the extruded PCL tubes, implants were fabricated with three different surface areas, as generated by varying the implant length: 82 ± 1 mm^2^, 311 ± 4 mm^2^_,_ and 543 ± 5 mm^2^, with an average of 31, 124, and 216 mg of TAF loaded into the implant, respectively. All devices comprised Sigma-PCL with a wall thickness of 100 µm, an OD of 2.5 mm, and a formulation of 2:1 TAF:castor oil. The cumulative release of TAF from the implants were monitored for approximately 30 days, as shown in Figure 2a. As expected for a membrane-controlled system, the higher surface area results in a higher release rate of TAF from the implant. Furthermore, the linear relationship between daily release rates and surface area supports the mechanism of membrane-controlled release from these implants (Figure 2b). These results align with a previous report using reservoir-style devices with thinner walls of PCL (8.5 µm) fabricated via solvent film-casting [55]. In the current study, devices were fabricated using PCL tubes prepared via melt extrusion, which produced thicker walled tubes (between 45–200 µm). Despite the thicker PCL wall and different fabrication approach, these devices also maintained membrane-controlled release in this range of wall thickness, demonstrating the robustness of the PCL-based drug delivery platform. For the remainder of this paper, the cylindrical geometry was fixed at 2.5 mm OD and 40 mm length to accommodate commercially available trocars utilized for contraceptive implants [56,57], and the release rates were normalized to the surface area of 314 mm^2^.

**Figure 3:**
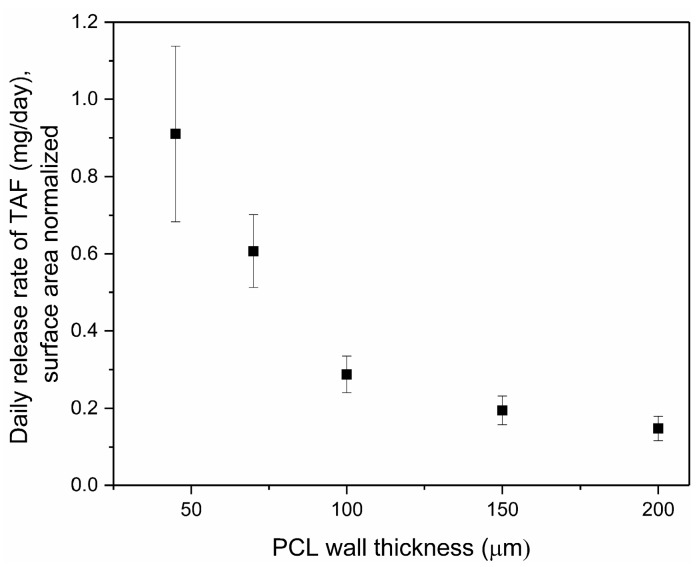
Daily release of TAF (mg/day) from implants with different wall thicknesses as calculated over 35 consecutive days within an in vitro assay. Implants comprised a 2:1 TAF-castor oil formulation (dimensions: 2.5 mm outer diameter (OD) by 40 mm length) fabricated with Sigma-Grade PCL. Three implants were tested per condition.

The thickness of the implant walls was another attribute that affected release rates of drug. Figure 3 shows the daily release rates of TAF from implants comprising PCL of different wall thicknesses and containing a formulation of 2:1 TAF:castor oil excipient. The release rates of TAF inversely correlated with thickness of the PCL walls: 0.91 ± 0.23 mg/day (45 µm), 0.61 ± 0.09 mg/day (70 µm), 0.29 ± 0.05 mg/day (100 µm), 0.19 ± 0.04 mg/day (150 µm), and 0.15 ± 0.03 mg/day (200 µm). As the wall thickness increased from 45 to 200 µm, the release rates approach a plateau wherein the release rates of TAF show minimal change. Importantly, the daily release rates were calculated over the first 35 days of TAF release from the implants, which included a burst release that is more pronounced in thinner walled implants that results in a higher standard deviation (e.g., 45 µm walled implant). We speculate the burst release may arise from transport of drug into the PCL. This inverse relationship between the thickness of the PCL walls and the release rates of TAF was also demonstrated for thin-walled PCL implants fabricated by a solvent casting approach despite considerable differences in the device processing technique [55]. To reserve adequate volume in the reservoir for drug load, this study only investigated wall thickness up to 200 µm. Overall, these experiments demonstrate the ability to employ two parameters, surface area or wall thickness, to tailor the release rates of TAF from a reservoir-style implant fabricated with extruded PCL tubes.

### 3.2. Effects of PCL Properties on Implant Performance

**Figure 4:**
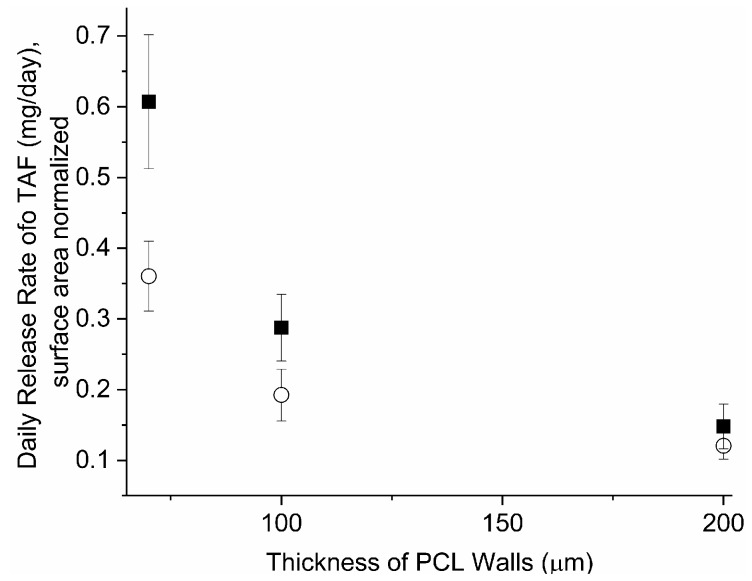
Effect of PCL type on daily release rates of TAF (mg/day) from implants with different wall thicknesses and fabricated with (■) Sigma -PCL or (○) PC-12. Implants contained a formulation of 2:1 TAF-castor Oil. Daily release rates were calculated from release over at 35 days and three implants were tested per condition.

PCL is a semi-crystalline, hydrophobic polymer with biodegradation kinetics that depend on the initial MW, typically occurring in the order of 1–2 years [38], which supports a LA PrEP implant. In these studies, PCL starting material with two different MWs were selected to potentially support an implant with target duration of 6–12 months: Sigma-PCL (*M*_n_ of 103 kDa) and PC-12 PCL (*M*_n_ of 51 kDa). PCL tubes of different wall thicknesses (70, 100, 200 µm) were extruded with either Sigma-PCL or PC-12 and subsequently filled with a formulation of 2:1 TAF:castor oil. Evaluation of these implants using in-vitro release assays revealed two important concepts (Figure 4). First, the release rates of drug from the implant depended on the selection of PCL; TAF releases at a higher rate from implants comprising Sigma-PCL as compared to implants comprising PC-12. Interestingly, the influence of PCL type on TAF release rates is minimal in tubes with thicker walls (e.g., 200 µm) versus thinner walls (e.g., 70 µm). Second, Figure 4 also shows that irrespective of the PCL type used to fabricate the implant, the release rates of TAF still scales inversely with wall thickness, as also shown in Figure 3.

**Figure 5:**
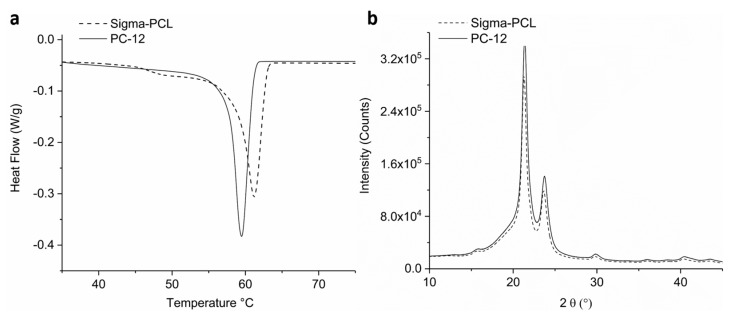
Exemplary graphs of (**a**) DSC heat flow curves and (**b**) XRD profiles of PCL tubes with 100 µm wall thickness.

It is possible that differences in crystallinity between the two types of PCL starting materials could affect the release rates of TAF from the implant. Therefore, to further understand the effect of polymer properties on release rates of drug, extruded tubes comprising PC-12 or Sigma-PCL were evaluated with DSC and XRD. Analysis by DSC showed that all PCL tubes exhibit a melting endotherm with a peak near 60 °C (Figure 5A, Figure S1), the characteristic melting temperature (*T_m_*) of PCL [58,59]. However, notable differences in the melting endotherms were also evident, such as a narrower melt transition of PC-12 compared to Sigma-PCL and the presence of a small shoulder peak around 50 °C in Sigma-PCL which was absent in PC-12. Quantitatively, the specific *T_m_* values also differed; Sigma-PCL showed a slightly higher *T_m_* compared to PC-12 for all thicknesses of the tube walls (Table 1 and Figure S1). For each sample, Equation (1) was used to calculate the mass % crystallinity and Equation (2) (Thompson–Gibbs equation) was used to calculate the crystallite sizes. Results in Table 1 show that irrespective of the wall thickness, the crystallite size of PC-12 was slightly lower than the crystallite size of Sigma-PCL. Moreover, the crystallite size of Sigma-PCL slightly varied with different tube thicknesses, whereas PC-12 remained consistent. The % crystallinity was slightly higher in certain cases for PC-12 compared to Sigma-PCL, showing statistically significant differences for extruded tubes with 70 and 200 µm wall thicknesses.

XRD analysis was also performed to further examine the crystallite size of PCL extruded tubes using the Scherrer equation (Equation (3)). Extruded tubes (100 µm wall thickness) fabricated from Sigma-PCL and PC-12 showed similar diffraction patterns that include intense Bragg peaks at 2θ near 21.3° and 23.7°, correlating to diffraction of the (110) and (200) planes of the PCL crystallite, respectively (Figure 5B) [60,61]. Results from XRD analysis (Table 2) show that the crystallite sizes of PC-12 were slightly smaller than Sigma-PCL, where Sigma-PCL total crystallite sizes was 25 nm (14.2 + 10.8) and PCL-12 was 23.4 nm (13.2 + 10.2), which also agrees with DSC data. Both techniques used to measure crystallite size indicate a similar order of magnitude from the two PCL types, therefore it is unlikely that crystal size alone was responsible for the differences in drug diffusion kinetics from the materials considered in this study, however the observation that crystallite size increased with tube thickness for Sigma-PCL (as measured by DSC) may play a role in release kinetics.

Taken together, these data highlight the importance in considering properties of drug transport in products comprising semi-crystalline polymers, which contain both amorphous regions amenable to drug transport when above the polymer glass transition temperature (*T*_g_), and crystalline regions which pose a diffusive mass transport barrier. These data indicate that PCL is an ideal polymer suited for membrane-controlled drug diffusion applications given its material properties and semi-crystalline nature. For example, PCL has a *T*_g_ of −60 °C which allows for drug transport at physiological conditions (37 °C) where the amorphous regions exhibit adequate free volume for passive diffusion of small molecules and fluid driven by concentration gradients. Concurrently, PCL crystals impart structural integrity to the implant and act as a transport barrier which modulate drug diffusion and allow for sustained release of TAF. The DSC and XRD results presented here suggest that crystallite size, quantity of crystallinity, and ultimately polymer free volume within PCL will impact transport properties of TAF through the polymer, as also supported by studies with other systems [62]. Our results show that extruded tubes with lower MW (PC-12) contain smaller sizes of crystals and slightly higher % crystallinity (statistically significant for 70 and 200 µm tubes, *p* = 0.008 and *p* = 0.007, respectively) as compared to PCL with higher MW (Sigma-PCL). This suggests that higher degree of crystallinity and smaller crystallites could create a more tortuous path for diffusion of the drug, leading to a lower release rate from the implant. At 37 °C, TAF likely diffuses through the amorphous regions of PCL, where the polymer exhibits greater segmental mobility to facilitate passage of small molecules. The size and quantity of the crystal regions would affect the spatial arrangement and quantity of these amorphous regions, ultimately affecting transport kinetics. These findings are supported by the mathematical relationship between membrane flux through a given area which is inversely proportional to distance traveled (wall thickness) by the constant of mass diffusivity, i.e., Fick’s first law of diffusion. The diffusion constant is a function of temperature, molecular size, and viscosity. For polymers, the viscosity term describes polymer free volume, which is impacted by crystallinity, hence the differences in material properties and resultant release rates were observed here.

In addition to the quantitative differences in polymer physicochemical properties observed in this study, three important qualitative findings are also of note. First, Sigma-PCL exhibited irregularities in the melting endotherm as evidenced by a small but apparent shoulder peak prior to the melt whereas PC-12 did not. It was likely a result of thermal history incurred in Sigma-PCL processing from the manufacturer. Given this consideration, the shoulder peak likely did not represent the PCL crystalline phase and may have contributed to an over-estimation of the crystalline content of the Sigma-PCL. A second difference was noted when comparing the width of the melt transition where PC-12 exhibited a narrower melt endotherm compared to Sigma-PCL, suggesting a tighter distribution of polymer molecular weight comprising the crystalline phase. Finally, another difference between these two grades of material was the crystallite size as a function of tube thicknesses. While PC-12 demonstrated more consistency in crystallite size, Sigma-PCL crystallite size increased with tube thickness, indicating a lack of control on the final properties during processing and perhaps explaining the large variability in release rates at low thicknesses and more consistent release rates at higher thicknesses. Sigma grade PCL also exhibited a steeper decline in release rate with respect to wall thickness, while PC-12 demonstrated a more gradual decline in release rate as a function of thickness. It was hypothesized that the concomitant increase in wall thickness and crystallite size observed with Sigma PCL was responsible for the attenuation in release rate values for the two grades of PCL at the higher wall thicknesses studied here. Taken together, these observations highlight the importance of material choices in the design of drug delivery devices from an engineering and quality control perspective.

### 3.3. Performance and Fabrication of a LA PCL Implant for Delivery of TAF 

The duration of this reservoir-style implant for TAF is dictated by two parameters: the drug quantity within the reservoir and the rate of drug release from the implant. Using selected implant dimensions (2.5 mm OD, 40 mm length), TAF payload within the reservoir for the 2:1 TAF:castor oil formulation was approximately 115 mg for an implant with wall thicknesses of 100 µm. Within these constraints of drug payload, the duration of a single TAF implant for PrEP ultimately depends on the daily drug release required for protection as administered via the subcutaneous route, which is currently unknown. In this manuscript, in-vitro release rates from prototype implants were tailored to the range between approximately 0.2 and 0.8 mg/day from a single device, as suggested from previous animal studies and in-silico modelling set with TFV-DP target concentrations of >48 fmol/10^6^ cells among 500 virtual healthly women [31,63]. 

**Figure 6:**
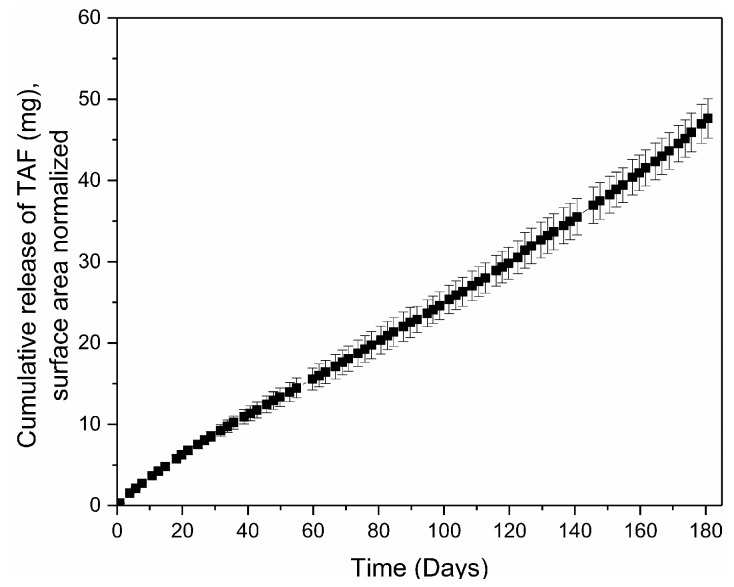
Cumulative release of TAF (mg) from an implant comprising Sigma-PCL of 100 µm wall thickness at 2.5 mm outer diameter and 40 mm length. Implants contained a 2:1 TAF-castor oil formulation. Three implants were tested per condition.

Using these dimensional parameters, a batchwise process was developed to fabricate TAF implants from extruded PCL tubes, which entails loading the drug formulation into the cavity of a PCL tube and sealing the ends. Given the low melting temperature of PCL (60 °C), the implant was readily end-sealed by controllably heating PCL into the desired geometry using an in-house customized polymer extruder. Care was given to avoid contamination of the interior walls of the tube near the sealing points, which could hinder the formation of end seals during the melt sealing. This fabrication process was used to generate implants for a six-month in-vitro study to assess the release of TAF. As shown in Figure 6, implants released TAF at a rate of 0.28 ± 0.06 mg/day over the course of 180 days. After 180 days, approximately 68 mg of TAF remained within the implant, with a chromatographic purity of 89.2 ± 0.8% (Table S1). The trend of decreased TAF stability over time results from ingress of water into the implant as drug depletes, which, in turn, facilitates hydrolytic degradation of TAF [64]. We are currently evaluating other formulations of TAF that help optimize stability. The implant maintained structural integrity throughout the 180-day release period in simulated physiological conditions.

To support in vivo use of the implant, gamma irradiation was used to sterilize the implant after fabrication. Since gamma irradiation has been shown to affect the chemical and physical properties of PCL [65,66], studies were performed to evaluate its potential effects on the implant performance. Table S2 shows the GPC analysis of PCL, including samples of PCL raw material used for the extrusion process and extruded PCL tubes before and after gamma irradiation. Both PCL types (Sigma-PCL and PC-12) showed a slight decrease in *M*_n_ after gamma irradiation, as expected, but the extrusion process minimally affected the *M*_n_ of PCL. To further ascertain release rates of TAF from the implant after gamma irradiation, in vitro release assays were performed on implants with and without gamma irradiation at dosages between 18–24 kGy. As shown in Table 3, the release rates were comparable irrespective of treatment with gamma irradiation and the difference in release rates were not statistically significant when comparing non-irradiated and gamma irradiated release rates (*p* = 0.27 and *p* = 0.42 for Sigma-PCL at 70 and 100 µm, respectively; *p* = 0.11 and *p* > 0.99 for PC-12 at 70 and 100 µm, respectively).

In summary, the in vitro studies presented in this manuscript demonstrate a new fabrication method to produce reservoir-style PCL implants using a batchwise process of polymer tube extrusion, formulation filling, and implant sealing via melt procedures. Although this fabrication process is not currently conducive to high-throughput manufacture, incremental steps detailed here support future production and implementation efforts. For instance, use of melt extrusion to produce PCL tubes presents an improvement over previously reported thin-film casting techniques, in terms of throughput of tube manufacturing and ultimate robustness of the implant. By increasing the implant wall thickness (e.g., from 70 to 200 µm) using an extrusion method, the implants were more aesthetically pleasing, exhibited greater mechanical sturdiness during handling and therapeutic use, while simultaneously retaining sustained release kinetics of drug. Moreover, the injection end-sealing method presented here produces an implant suitable for commercially available trocars, and is loaded with enough of the drug to last longer than six months; all considerations that were deemed preferable by end-users [67,68]. With an eye towards product development needs for clinical implementation, compatibility with applicators already clinically employed offers options to adopt existing procedures to support LA-PrEP rollout.

## 4. Conclusions

As the field of HIV PrEP rapidly progresses, new LA drug delivery systems must respond to evolving clinical and socio-behavioral insights in terms of effective ARV dosing, desired duration, and product acceptability. This paper describes a reservoir-style implant with the flexibility to adapt to needs of this advancing field by readily tailoring properties of the implant, including surface area, wall thickness, and properties of PCL. The first section of this manuscript describes the ability to control release rates of TAF via dimensions of extruded PCL tubes (i.e., surface area, wall thickness). We report an inverse correlation between wall thickness and drug release rates and this feature was further used to tune TAF dosing between 0.91 ± 0.23 mg/day (45 µm wall thickness) and 0.15 ± 0.03 mg/day (200 µm wall thickness). Our studies further show that the release rates of TAF scale proportionally with the surface area of the implant, demonstrating the membrane-controlled release from extruded PCL tubes. These results build from previous work using implants made of thin-films of PCL, which reported similar trends with the ability to tune release rates from reservoir-style devices between 8 and 15 µm thick [55]. However, the replacement of solvent-cast thin films with extruded tubes, as described here, supports the development of future manufacturing processes and ultimately provides robust implants with thicker walls for greater ease of handling. Although outside the scope of this manuscript, additional strategies to further increase drug stability, dosing and duration from a single implant, while simultaneously maintaining compatibility with market-available trocars, include modifications to the formulations such as selection and quantity of excipient. Although in vivo studies were not reported in this paper, the in vitro assay conditions used here have shown good in vitro/in vivo correlations previously [57]. Future in vivo studies will further advance this implant technology.

We also described a batchwise fabrication of implants using two types of PCL starting materials, either PC-12 or Sigma-PCL. In these studies, the selection of PCL starting material affects the release rates of TAF from the implant; TAF releases faster from implants fabricated with Sigma-PCL as compared to PC-12. Analysis via DSC and XRD were used to probe the reason for these differences. Analysis by DSC revealed slight differences in the mass percent of crystallinity from both PCL types (70 and 200 µm wall thickness) and showed slight differences in the crystalline sizes. Although additional studies are needed to further characterize these differences, our results do suggest that a smaller crystalline size and higher mass % crystallinity within the polymeric material retard the diffusion of TAF through PCL. Interestingly, when using these thicker walled tubes (e.g., 200 µm walls), the effect of PCL type on release rate is less pronounced. Using down-selected parameters, we demonstrate delivery of TAF in vitro for 180 days at a dose of 0.28 ± 0.06 mg/day and the TAF within the implant maintains stability at 89.2% ± 0.8% at the end of this duration. This is the first report of a six-month in vitro study showing sustained release of TAF from a biodegradable trocar-compatible implant. This manuscript also shows that the use of gamma irradiation (dosages 18–24 kGy) as a sterilization technique minimally affects the release rates of TAF, which benefits future in vivo studies. Overall, this implantable drug delivery system holds various parameters that can be tuned to achieve a targeted dose of TAF. Although the final design of this drug delivery system for TAF awaits feedback from dosing requirements, these promising results help to highlight the path towards the goal of developing LA delivery of TAF for HIV PrEP.

## Figures and Tables

**Table 1:** Thermal properties of PCL extruded tubes from DSC analysis.

PCL Type	Wall Thickness (µm)	*T_m_* (°C)	% Crystallinity	Crystallite Size (nm)
PC-12	70	59.4 ± 0.1	56 ± 1.0	27 ± 0.2
	100	59.4 ± 0.1	53 ± 2.0	27 ± 0.4
	200	59.7 ± 0.4	56 ± 1.0	27 ± 1.2
Sigma-PCL	70	60.7 ± 0.1	53 ± 0.3	31 ± 0.2
	100	61.1 ± 0.2	52 ± 1.2	32 ± 0.6
	200	61.3 ± 0.1	53 ± 0.1	33 ± 0.3

**Table 2:** Thermal properties of PCL tubes * from XRD analysis.

PCL Type	Crystallite Size (nm)	
	*L*_110_	*L*_200_
PC-12	13.2	10.2
Sigma-PCL	14.2	10.8

* Extruded tubes comprised 100 µm wall thickness.

**Table 3:** Daily TAF release rates from implants pre- and post-gamma irradiation.

PCL Type	Wall Thickness (µm)	Release Rates of TAF (mg/Day)	
		Non-Irradiated	Gamma Irradiated
Sigma-PCL	70	0.62 ± 0.09	0.54 ± 0.06
	100	0.29 ± 0.05	0.32 ± 0.03
PC-12	70	0.37 ± 0.05	0.30 ± 0.03
	100	0.20 ± 0.03	0.20 ± 0.02

Formulation of 2:1, TAF:castor oil; Daily release rates calculated from 30 days of consecutive release.

## Supplementary Materials

The following are available online at https://www.mdpi.com/1999-4923/11/7/315/s1. Figure S1: Heat flow versus temperature for extruded tubes comprising (**A**) Sigma-PCL and (**B**) PC-12 at different tube thicknesses, Table S1: Stability of TAF in implants at different timepoints within in vitro conditions, Table S2: GPC Analysis of PCL.
