# Supplementary material for: Characterization of a Reservoir-Style Implant for Sustained Release of Tenofovir Alafenamide (TAF) for HIV Pre-Exposure Prophylaxis (PrEP)
Source: Pharmaceutics. 2019 Jul 4;11(7):315. doi: 10.3390/pharmaceutics11070315 (PMC6680758; doi:10.3390/pharmaceutics11070315)
Supplement: Supplementary file 1 [file pharmaceutics-11-00315-s001.pdf]

# Supplementary Materials: Characterization of a Reservoir-Style Implant for Sustained Release of Tenofovir Alafenamide (TAF) for HIV Pre-Exposure Prophylaxis (PrEP)

**Leah M. Johnson *, Sai Archana Krovi, Linying Li, Natalie Girouard, Zach R. Demkovich, Daniel Myers, Ben Creelman and Ariane van der Straten**

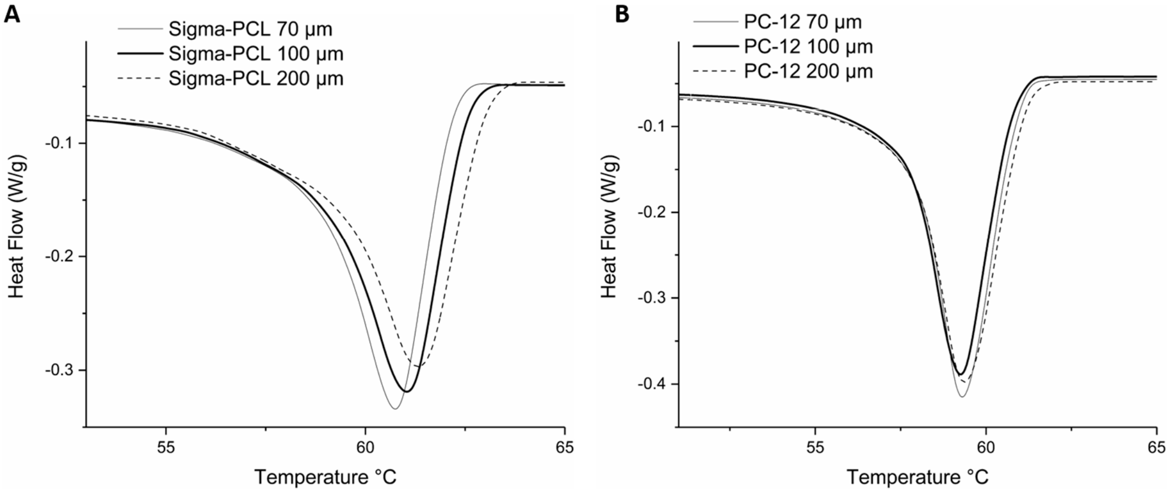


**Figure S1.** Heat flow versus temperature for extruded tubes comprising (**A**) Sigma-PCL and (**B**) PC-12 at different tube thicknesses.

**Table S1.** Stability of TAF in implants at different timepoints within in vitro conditions *.

| Days | % Stability |
|---|---|
| 90 | 97.1 ± 0.09 |
| 120 | 96.5 ± 0.17 |
| 15 | 92.5 ± 0.53 |
| 180 | 89.2 ± 0.84 |

* Implants comprise Sigma-PCL of 100 μm wall thickness, 2.5 mm OD, and 40 mm length containing a 2:1 TAF-Castor Oil formulation. Implants were not gamma-irradiated.

**Table S2.** GPC Analysis of PCL.

| Type of PCL | | Sigma-PCL | PC-12 |
|---|---|---|---|
| PCL raw material without gamma irradiation | $M_n$ (kDa) | 103 | 51 |
| | $M_w$ (kDa) | 148 | 89 |
| PCL extruded tubes * without gamma irradiation | $M_n$ (kDa) | 98 | 52 |
| | $M_w$ (kDa) | 145 | 88 |
| PCL extruded tubes * after gamma irradiation | $M_n$ (kDa) | 70 | 41 |
| | $M_w$ (kDa) | 119 | 77 |

* Wall thickness of PCL tubes of 70 μm
